# A new method for training creativity: narrative as an alternative to divergent thinking

**DOI:** 10.1111/nyas.14763

**Published:** 2022-03-10

**Authors:** Angus Fletcher, Mike Benveniste

**Affiliations:** ^1^ Project Narrative The Ohio State University Columbus Ohio

**Keywords:** creativity, narrative, divergent thinking, innovation, causal thinking, artificial intelligence

## Abstract

Creativity is a major source of innovation, growth, adaptability, and psychological resilience, making it a top priority of governments, global corporations, educational institutions, and other organizations that collectively invest hundreds of millions of dollars annually into training. The current foundation of creativity training is the technique known as divergent thinking; yet for decades, concerns have been raised about the adequacy of divergent thinking: it is incongruent with the creative processes of children and most adult creatives, and it has failed to yield expected downstream results in creative production. In this article, we present an alternative approach to creativity training, based in neural processes different from those involved in divergent thinking and drawing upon a previously unused resource for creativity research: narrative theory. We outline a narrative theory of creativity training; illustrate with examples of training and assessment from our ongoing work with the U.S. Department of Defense, Fortune 50 companies, and graduate and professional schools; and explain how the theory can help fill prominent lacunae and gaps in existing creativity research, including the creativity of children, the psychological mechanisms of scientific and technological innovation, and the failure of computer artificial intelligence to replicate human creativity.

Here's a paradox: according to current research, young children are more imaginatively creative than adults^1^, ^2^, ^3^, ^4^, ^5^, ^6^, ^7^, ^8^, ^9^, ^10^, ^11^, ^12^, ^13^, ^14^, ^15^—yet, also according to current research, creativity's main neural engine is divergent thinking, which relies on memory and logical association, two tasks at which young children underperform adults.^10^, ^16^, ^17^, ^18^, ^19^, ^20^, ^21^, ^22^, ^23^, ^24^, ^25^

This is not an idle puzzle. Creativity is a major source of innovation, growth, adaptability, and resilience, making it a top priority of governments, global corporations, kindergarten‐through‐professional educational institutions, and other organizations and individuals who collectively invest hundreds of millions of dollars annually into training in divergent thinking and related practices (e.g., combinatorial play, associational fluency, analogical reasoning, multiuse sets, design schooling, brainstorming, and innovation forecasting).^19^, ^21^, ^26^, ^27^, ^28^, ^29^, ^30^, ^31^, ^32^, ^33^, ^34^, ^35^, ^36^, ^37^, ^38^, ^39^, ^40^ Yet, for all the tangible gains that training has yielded, its incongruity with children's mental processes raises a question:^22^, ^41^ How much more could be gained if the focus was placed instead on the alternative, creative engine that young minds employ?

In this article, we will suggest that quite a bit can be gained, and we will propose a method for gaining it. Our starting point will be that the above paradox reflects modern creativity training's overwhelming emphasis on computation, an emphasis that has had the great merit of demystifying creativity and rendering it transparently teachable and assessable, but that has also led researchers to downplay the fact that the human brain runs noncomputational processes that are as mechanical—and therefore, as teachable and assessable—as memory and logic.^42^, ^43^ One such process is narrative cognition (i.e., thinking in actions, causes, and outcomes). Narrative cognition has long been known to play a role in creativity,^44^, ^45^, ^46^ but modern creativity's computational focus has relegated that role to postgenerative stages, such as convergent thinking.^47^, ^48^, ^49^, ^50^, ^51^, ^52^ This, we believe, is unnecessarily limiting: narrative cognition is a core driver of the brain's ability to conceive original actions and, therefore, to invent technologies, do new science, make story‐based art (such as novels and films), and solve problems. And, as research in narrative theory has demonstrated, and our own work with schools, government organizations, and corporations is affirming, narrative cognition is trainable, throwing open the door to a new curriculum that can enrich students of all ages and professions with more of the nonlogical creativity observed in the young.^53^, ^54^, ^55^, ^56^, ^57^, ^58^, ^59^, ^60^

To make the case for this broad expansion of creativity training, the following pages will present a narrative theory of creativity; describe how the theory can be operationalized into a suite of practical techniques for training government leaders, corporate executives, and students; and detail how the theory closes gaps and reconciles apparent contradictions in prior creativity research. Our purpose in advancing this new theory is not to refute divergent thinking and other computational methods of training creativity; rather, it is simply to establish their insufficiency, clearing space for innovation. To clear that space, however, it is necessary to differentiate our narrative approach from computational ones, so we will begin with two preliminary sections, the first to provide a narrative definition of creativity, and the second to delineate the theoretical assumptions that have led current creativity researchers to place so much emphasis upon divergent thinking.

## Defining creativity: just novel or also useful?

Creativity has a long folk history of being viewed as an ineffable, even supernatural power, but over the past 80 years, researchers in creativity studies have worked to reduce it to quantifiable factors. Those factors are, however, currently disputed. The standard definition is that creativity is the ability to generate novel ideas that are useful.^61^, ^62^, ^63^ This majority view reflects creativity's valuation in business and most social contexts as a source of problem solving, innovation, and other practical outcomes, yet it has been contested on the grounds that utility can be incidental, irrelevant, and even in tension with creativity: many scholars of creativity treat utility as less foundational than originality and its attendant qualities (e.g., surprise);^64^, ^65^ most nonacademics perceive creativity as almost entirely synonymous with novelty;^66^, ^67^, ^68^ and practical experience suggests that too much emphasis on practicality can inhibit the creative process, promoting minor, short‐term improvement at the expense of major, long‐term innovation. The alternate view, therefore, is that creativity is mostly, or entirely, reducible to the ability to generate novel ideas.^69^, ^70^, ^71^

Both sides of this debate have contributed to creativity research, and practically speaking, the more that their competing concerns can be integrated, the more fruitful that creativity training can be. Such integration can be facilitated in part by a narrative approach, because where current definitions of creativity (both the standard and alternative views) treat cognition as a means to generate *ideas* (and, therefore, models, relations, representations, and patterns), narrative cognition is a biological mechanism for generating *actions* (and, therefore, effects). Narrative cognition's evolved purpose is thus physical function; so, a narrative approach to creativity tends toward useful outcomes without requiring an explicit emphasis on utility, offering a way to keep creativity training free from the inhibitions, pressures, and constraints imposed by the insistence that the training results in obviously practical applications, while, nevertheless, answering the demand that corporations, schools, and other institutions typically invest in creativity training because they are seeking practical results. Stated definitionally, a narrative approach distinguishes creativity from innovation while linking creativity to innovation as its precursor, so it treats creativity as *experimental action* and innovation as *creativity that succeeds*.

## Why current creativity training emphasizes divergent thinking

The breakthrough in modern creativity training came in the 1950s from the research of J. P. Guilford. Guilford had worked as a psychometrician for the Air Force during World War Two, leading him to conclude that a swathe of intellectual capacities—including creativity—not captured by standard IQ tests could be quantified in other ways.^72^ Out of those efforts at quantification, Guilford developed his Structure of Intellect theory,^73^ an intricate construct that led to many influential ideas about creativity, the most prominent being *divergent production*, or more commonly, divergent thinking.^72^, ^74^

Divergent thinking is a computational process of spatial, logico‐semantic combination. It derives from Guilford's view of the human brain as a sense‐making information processor that absorbs data through the senses, stores it in memory (in the form of figural, semantic, and symbolic content), and processes it via logical protocols (e.g., deduction, inference, association, and analogy).^74^, ^75^ That view of the brain enabled Guilford to systematize creativity into a rational science that was clear, teachable, and assessable; so, even though the Structure of Intellect theory was dismissed by later psychologists as an “eccentric aberration,”^73^ Guilford's general commitment to reducing creativity to a set of logical tasks that ran on symbolic data was adopted in the 1950s, 1960s, and 1970s by Alex F. Osborn (the inventor of brainstorming), Sarnoff Medick (the author of the Remote Associations Test), William J. J. Gordon (an advocate of conceptual transposition and defamiliarization), and D.T. Campbell (a theorist of blind variation and selective retention) and has, over the past half‐century, become foundational to the academic field of creativity studies and its most well‐known subfield, design.^76^, ^77^, ^78^, ^79^, ^80^

This computational approach to analyzing and measuring creativity has had many practical gains in both creativity training and assessment. In training, it has led to exercises that increase divergent thinking by expanding working memory, fostering analogical thinking, improving associational fluency, promoting diverse mix‐and‐matching from mental sets, nurturing combinatorial play, and leveraging brainstorming into focused output via convergent thinking, critical thinking, causal winnowing, and problem solving.^16^, ^19^, ^20^, ^21^ In assessment, it has yielded tests for measuring the quantity and diversity of creative ideas.^26^, ^81^, ^82^, ^83^, ^84^, ^85^, ^86^, ^87^, ^88^ The most well‐known and widely used of those tests is the Torrance Test, which was invented in the 1960s and incorporates many of Guilford's original assessment tools (including Unusual Uses, the Impossibilities Task, the Consequences Task, the Improvement Task, the Common Problems Task, and the Situations Task), while also adding a number of pictorial assessments.^89^, ^90^

These tests and exercises became over the later 20th century, and remain in our 21st century, almost the entirety of creativity training within the American higher education system (from middle school to graduate design), global business (across all sectors, from manufacturing to finance to tech to services), and the U.S. government (from the Department of Education to the military's special operations community).^91^, ^92^, ^93^, ^94^, ^95^, ^96^, ^97^ But even as they have produced significant, measurable improvements in divergent thinking, they have not yielded the expected gains in downstream outcomes, such as innovation, growth, and resilience.^92^, ^98^, ^99^, ^100^, ^101^, ^102^, ^103^ This disjunction has prompted multiple attempted fixes, each of which has doubled‐down in a different way on Guilford's belief that creativity can be reduced to computational processes. One fix, championed by psychologists, including Michael D. Mumford, is more effective training in divergent thinking's logical partner: convergent thinking.^29^, ^35^, ^49^, ^51^, ^104^, ^105^ A second fix, championed by organizational researchers, including Adam Grant and Jeff DeGraff, is to better operationalize divergent thinking via behavioral and management strategies such as intentional procrastination and the Innovation Code.^106^, ^107^, ^108^, ^109^, ^110^, ^111^, ^112^, ^113^ A third fix, increasingly popular among design scholars, is to disrupt fixation bias and cognitive inflexibility by introducing convergent and critical thinking into earlier stages of the idea generation process.^49^, ^50^, ^114^ A fourth fix, advocated by innovation specialists John and Markus Baer, is to narrow brainstorming on more precisely defined problems and to engage more domain‐specific knowledge.^28^, ^99^, ^115^

These interventions have yielded practical gains, but they have all sidestepped the possibility that divergent thinking's unexpectedly low returns might evince a limit of divergent thinking itself.^42^, ^43^, ^99^, ^102^, ^103^, ^116^, ^117^ That such a line of inquiry might be nonobvious, unappealing, or even incoherent to modern creativity researchers is understandable, given that how indebted modern creativity studies and design are to the notion of divergent thinking. By convincing broad sectors of government, industry, and education that creativity and innovation can be systematically implemented, even to the point of automation, Guilford's elegant distillation of idea generation to a data‐driven, logical process of semantic combination, associative play, and transpositional analogy has become the base upon which the apparatus of modern creativity training rests. But does the fact that creativity *can* be logical mean that creativity is *only* logical? Are child and adult creatives primarily notable for their logical aptitude? Are individuals with advanced logical skills more likely to be successful creatives? No. And in fact the opposite,^1^, ^7^, ^22^, ^118^, ^119^, ^120^, ^121^, ^122^ suggesting that one way to improve the downstream results of creativity training would be by enlarging upstream methods of creative generation with nonlogical but mechanical sources of original thinking, of which a promising candidate is narrative.

## A theory of narrative creativity

The human brain can cogitate in more than logical rules, symbols, bytes, representations, and other computational methods and materials. It can cogitate in action. Action is composed of a cause and its effect. Action causally sequenced to another action (or actions) is narrative.

Narrative has been incorporated into prior creativity training for adults, but the training has limited narrative (and its various components and constructs, including causal thinking) to forecasting, convergent thinking, and other late‐stage, postgenerative processes, rejecting (either tacitly or explicitly) the possibility that narrative cognition can be a *source* of original plans, strategies, and so on.^48^, ^49^, ^51^, ^123^ This limited role for narrative cognition in current creativity training reflects Guilford's belief that human intelligence is entirely computational. Guilford was not the originator of this belief about human intelligence; it was popularized via an influential 1943 paper by Warren McCulloch and Walter Pitts and is traceable backward through Gottlob Frege, to George Boole, to Thomas Hobbes, to Aristotle's *Organon*.^124^ But the belief became increasingly popular after Guilford, partly because of Guilford's role as an originator of modern creativity studies, and partly because of the rise of modern AI (which counts McColloch and Pitts as founding figures). Over the previous half‐century, computational approaches to human cognition have been adopted by many computer scientists, cognitive scientists, and psychologists,^125^, ^126^ and they have prompted creativity researchers to treat narrative as though it too is computational, reducible to the semantic processing and association of representational and symbolic content.^26^, ^64^, ^127^, ^128^, ^129^, ^130^

This computational view of narrative has been disputed by decades of work in narrative theory. The challenge comes in various forms, the streamlined version being:

1. Computation operates via mathematical and logical equations that exist in an eternal present tense (e.g., 1 + 1 = 2 and “Bob is that man over there”).
2. The present tense cannot comprehend narrative because (a) narrative is composed of causes and their effects and (b) a cause must temporally precede its effect. If the cause is rendered in the present tense, the effect thus must be rendered in the future; if the effect is rendered in the present, the cause must be rendered in the past.
3. When presented with narrative, computation is, therefore, forced into a contradiction: it must simultaneously process two entities that cannot coexist. In response to this contradiction, logicians, and computer scientists—Aristotle and Judea Pearl, to name two—have developed workarounds that allow computation to extract semantic information from narratives, but the workarounds all involve methods of translating causation into correlation (e.g., *Aristotle walks* is transformed into *Aristotle is walking*, and then *Aristotle* is placed in a subject set tagged “cause,” while *walking* is placed in a predicate set tagged “effect”). The net consequence is to equate causes with their effects, such that causes are transformed from temporally prior sources of their effects into timeless signs of their effects. Fire, in other words, becomes a symbol (fire) that signals the necessary existence of smoke, rather than a material action that produces smoke. As a result, the physical processes involved in action are lost, deleting the causal mechanisms that makes narrative into narrative.
4. The same contradiction recurs when computation attempts to generate narrative: it is forced to render causation as correlation, thereby making action symbolic, distorting physics into semiotics, and replicating the magical thinking of late medieval science.^131^, ^132^

In our day and age, when computation is the most well‐articulated and pervasive analogy for reductionist models of human cognition, the claim that narrative is a mental act that cannot be computed may seem a prelude to invoking emergent properties, consciousness, or even the ineffable. But many mechanical problem‐solving processes (e.g., sawing, hammering, wrenching, and screwing) are noncomputational, and the same goes for narrative cognition's process of thinking from causes to effects. The full intricacies of the brain machinery involved in the latter process are still being uncovered, but they can be traced down to the level of the neuron.^133^ The neuron evolved roughly 550 million years ago as a device for generating behaviors that could help animal life acquire food and evade dangers,^134^ and some (and perhaps even at the beginning, all) of those behaviors were generated via a trial‐and‐error approach in which the neuron insentiently pulsed out action frequencies that it modulated in response to feedback.^135^, ^136^ That neuron‐instantiated trial‐and‐error mechanism was an elaboration of the creative engine of evolution by natural selection: it blindly generated original functions that were winnowed through use. In this, it operated very differently from Guilford's sense‐making computer, which proceeded not by generating narrative action sequences to see what worked but by inducting environmental information statistically for intentional decision making.^137^

This epistemic difference between neuronal trial‐and‐error and logical induction can seem to indicate that the former is less “intelligent” than the latter, and in the decades since Guilford began his research, many corporate, academic, and governmental thinkers (e.g., proponents of rational choice theory, general artificial intelligence, and quant‐based approaches to economic policy) have claimed that narrative‐driven behavior is inherently inferior to the data‐driven decision making of computers.^138^, ^139^, ^140^, ^141^ Such wholesale rejections of narrative cognition are belied, however, by the neuron's broader evolutionary history.^142^ Over that history, the neuron evolved not only a mechanism of spontaneous action but also an input–output mechanism guided by sensory organs, such as the eye,^143^, ^144^ eventually leading (through many evolutionary twists and turns) to the development of the animal visual cortex.^145^, ^146^ The visual cortex operates computationally, inducting data that are then employed to represent reality, and this computational capacity has proved so useful to life that it has propagated to other regions of the human neocortex, investing them with an ability to do math and perform logic.^147^, ^148^, ^149^, ^150^ Yet, as useful as computation is to the human brain, it has not totally supplanted the neuronal mechanism of experimental action, which remains entrenched in regions of the neocortex involved in generating and directing behavior.^151^ Hence, it is that the neurons of those regions pulse with random activity, as opposed to operating like mini‐addition machines that wait patiently for outside input.^152^, ^153^ Hence, it is that the human mind exhibits restless energy: anxiety, boredom, and split motivations.^154^, ^155^ Hence, it is that the brain can blindly generate new narratives, as, for example, the spontaneously original movement sequences of athletes and performers.^156^, ^157^ And hence, it is that even the visual cortex contains nonlogical activity that is gradually refined into more logical perceptions.^158^

Why did the brain evolve this way? Why did its computational powers not obsolesce its narrative activity? Why did natural selection not push the brain completely into data‐driven truth representation, rather than retaining a nonintentional mechanic of action generation? The likely answer is that nonintentional action has enormous practical utility in volatile, uncertain environments.^159^ In such environments, the data required for accurate computation are often absent, and even when it does exist, that data can only reveal which old actions worked in familiar yesterdays, not which new ones might work in unprecedented tomorrows.^160^ Such new actions must be generated by breaking from history, a breaking facilitated by the low‐data (even no‐data), thrash‐then‐adjust‐to‐feedback mechanism of archaic motor neurons. This, one can hypothesize, is why hundreds of millions of years of natural selection have not unilaterally preferred computational over narrative machinery. And indeed, such is the adaptive utility of the brain's narrative machinery that it can outperform the computational circuits of not only the human brain but also modern AI. Modern AI is much faster and more data‐intensive than the human brain. Yet, it is extremely fragile in unstable and uncertain environments because although computation is effective at revealing what behaviors correlate with success in previous contexts, it cannot generate and refine original actions in response to emergent challenges and opportunities—as the human brain's narrative machinery can.^161^

Narrative's adaptive utility has not gone unnoticed in current creativity research, which emphasizes the importance of trial‐and‐error in winnowing the downstream products of divergent thinking.^162^ But because creativity research's computational focus on divergent thinking has led it to neglect experimental narration in upstream activities, such as brainstorming, it has bypassed a major engine through which the adult brain invents original behaviors. As a result, it has not developed practical training methods that target the narrative mechanisms that the brain evolved to problem solve, grow, and innovate. To remedy this omission and better hone and strengthen the brain's creative capacity, a new approach must be ventured, one that helps human minds generate a richer and more flexible catalogue of causal actors and actions. And while that approach cannot be derived from logic, it can be derived from narrative theory.

## Narrative methods for increasing creativity

Narrative theory was birthed in 335 BCE by Aristotle in his *Poetics* (roughly a decade after his *Organon*) and has been empirically updated and expanded by modern scholars from R. S. Crane to James Phelan, leading to its adoption by thousands of global researchers through professional organizations, such as the International Society for the Study of Narrative, which comprehends fields from literature, to politics, to engineering, to the biological sciences.^163^, ^164^, ^165^ Narrative theory's object of inquiry is narrative art, that is, the historical catalogue of tools invented by storytellers to generate sequences of cause‐and‐effect in audience minds. Those tools reveal a great deal about narrative's mechanics, so even though the biological subtleties of narrative cognition are still being discerned, narrative theory can be used to devise new creativity training and assessment techniques that methodically target the brain's physiological processes for generating original action.

To begin mapping and measuring the practical yields of such an approach, we (the authors of this article) are collaborating with teams at the U.S. Army's Command and General Staff College, the U.S. military's special operations community, the University of Chicago Booth School of Business, the Ohio State College of Engineering, and other partners to implement and refine a creativity curriculum for senior military officers, corporate executives, and graduate students in fields from Entrepreneurship to the Arts.^a^ The curriculum is not meant to be prescriptive or to limit the ingenuity of future researchers; it is simply meant to serve as a model that demonstrates the general feasibility of mobilizing narrative theory to develop new creativity training.

The new training can, for organizational clarity, be subdivided into three categories of narrative technique: world building, perspective shifting, and action generating. All three categories can be found across global literatures, from ancient times to the present,^170^ a prevalence that may evince literature's adaptive function, but even if not, reflects literature's status as an organic development of the human brain's evolved priorities.^133^ Those priorities include an attention to new places (as sources of environmental threats and prospects), to new people (as competitors and allies), and to new events (as potential dangers and opportunities). So, it is that literature has historically captured audience interest by translating these three natural sources of attention into storyworlds, characters, and plotlines, respectively. The first uses narrative techniques to help the mind imagine new environments; the second, to help the mind imagine from different perspectives; and the third, to help the mind imagine possible future actions.

### World‐building techniques

In narrative literature, world building is often achieved by focalizing attention on a novel causal agent (e.g., an unexpected environmental event or actor) that prompts the audience's minds to hypothesize new possibilities for action. For example, if a narrative begins with an enchanted storm or a person flying, the audience's minds will speculate: *In this world, magic can happen*; or *in this world, humans can defy gravity*. We have translated this technique into creativity training in our work with the U.S. Army and the special operations community, where we have instructed participants to (1) identify unique events and actors in their operational domain and then (2) conjecture what unprecedented threats or opportunities those events and actors might portend. For example, in one training session, participants world built by imagining: *Autocratic regimes have devised new techniques for falsifying, hacking, and otherwise compromising data. What do these techniques reveal about the rules of how soft power operates in the modern world? How could those rules be leveraged into opportunities for developing new sources of democracy?* or *New virtual reality (VR) training devices have been discovered to undermine tacit knowledge transfer and other benefits of physical world pedagogy. What does this reveal about the rules of how pedagogy works in non‐VR environments? How could those rules be used to improve future VR training devices and curricula?*

### Perspective‐shifting techniques

In narrative literature, perspective shifting is often achieved by presenting the motives of a character or narrator, thereby allowing the audience to hypothesize how the character or narrator might act in a novel situation. We have translated this technique into creativity training at Fortune 50 companies, where we (1) pair executives with a partner, (2) ask each executive to solve a problem and then explain their problem‐solving motive (i.e., their causal thinking) to their partner, and (3) ask each executive to solve a second problem *using the motive of their partner*. For example, in one session, an executive at a consulting firm solved a case study on “the problem of improving service time at a 300‐shop auto center” by proposing that “a counterintuitive way to boost organizational efficiency is not to consolidate workflows but to decentralize them.” The executive's partner was then invited to apply this line of causal reasoning to other case studies that included “increasing a hedge fund's performance on nonstock assets” and “launching a media studio in an emerging market.”

### Action‐generating techniques

In narrative literature, action generation is often achieved by colliding two causal agents (e.g., two characters with different motives, or a character who opposes a rule of her environment) to produce a plot. We have translated this technique into creativity training for graduate students in fields from creative writing, to engineering, to business by asking them to speculate on unexpected events that could be prompted by the introduction of a new actor into a known environment—or by the introduction of a known actor into a new environment. For example, students generated and worked through counterfactual scenarios, such as: *What actions might Indra Nooyi take if she was placed in charge of the Department of Veterans Affairs?* and *What might Rachel Carson do if she woke up in a tomorrow in which carbon capture had reversed global warming?*

Further details on these narrative training techniques, along with dozens of others, are publicly available in a workbook prepared in 2021 for the U.S. Army.^171^

In the realm of assessment, this research has also yielded a new tool for measuring creativity. The tool is a modified version of the Consensual Assessment Technique (CAT).^172^ In the original CAT, expert judges are asked to rank how original and useful an idea is.^173^ In our modified version, expert judges are asked to rank how certain they are that an original action will work.^174^ This shifts the focus from creative ideas onto creative actions, and it also mitigates the problem of expert bias. Expert bias is a major feature of traditional approaches to creativity training because those approaches rely on logic, which in turn relies on data, and so on past performance. Experts are thus likely to overvalue ideas that would succeed in known contexts and undervalue ones that exploit emergent opportunities.^175^ Our modified CAT reduces this bias by associating creativity with expert uncertainty, taking advantage of expert experience to identify actions that are original enough to lie beyond the knowledge of veteran professionals.^176^, ^177^

## How a narrative approach can address gaps and contradictions in current research

Current creativity research is marked by several prominent lacunae and internal incongruities, including:

1. The most oft‐cited body of research on improving creativity in children is Anna Craft's work on “possibility thinking,” which proposes that counterfactual what‐if mental narratives can spur innovation via an action‐orientation that enables everyday problem solving. Yet, there is no theoretical work that explains the particular effectiveness of Craft's training method or translates it to adult contexts.^53^, ^54^, ^55^, ^56^, ^57^, ^58^, ^60^, ^120^, ^178^, ^179^, ^180^
2. Alison Gopnik's widely cited research demonstrates that children's story‐based imaginative activities (e.g., pretend play and pretense) constitute varieties of counterfactual exploration and facilitate causal learning.^3^, ^181^, ^182^ Yet, the neural mechanisms beneath these observed phenomena remain unclear.^183^
3. Advocates of divergent thinking have been forced to explain away children's creativity by redefining creativity to mean not just originality but utility, conflating creativity with innovation and narrowing innovation's operational range to conform to short‐term expert bias.^6^, ^64^, ^184^, ^185^
4. The Torrance Test assesses several kinds of narrative creativity (such as consequences and counterfactual thinking), yet current creativity training adheres mostly (and in adults, almost exclusively) to nonnarrative, logical processes.^16^, ^19^, ^20^, ^21^
5. On the Torrance Test, the Consequences and the Alternative Uses tasks are treated by current creativity researchers as varieties of divergent thinking. Yet, recent studies have shown that these tasks have different mental performance characteristics than associative tasks.^81^, ^186^
6. The ability of children to perform creative tasks drops after 4 or 5 years of schooling. Yet, that schooling is intensive in logical, semantic, and memory training.^24^, ^25^, ^118^, ^119^, ^120^, ^187^, ^188^, ^189^, ^190^
7. Neuroimaging has revealed that practical creativity requires more than memory and related semantic functions, yet those other functions have not been identified.^186^, ^191^, ^192^, ^193^
8. Scholars of the scientific method, from John Herschel, to Karl Popper, to Nancy Nersessian, to Lorenzo Magnani, have pointed out that novel scientific hypotheses depend on narrative leaps, creative intuitions, causal speculations, and abductive inferences, none of which can be reduced to semantic blending, transposition, or other computational procedures.^161^, ^191^, ^192^, ^194^, ^195^
9. Data are required for divergent thinking, so computational theories of mind treat data (accessed through the senses and memory) as critical for creativity. Yet, creativity is, nonetheless, possible in volatile and uncertain domains, where data are fragile or nonexistent.^192^, ^196^
10. According to the mechanics of divergent thinking, more data are always better for creativity. Yet, in practice, too much structured data impede creativity, which is why experts often struggle to be innovative and why children can be more imaginative than adults.^22^, ^31^, ^121^, ^122^, ^197^, ^198^, ^199^, ^200^, ^201^, ^202^
11. The major observed role of data in human creativity seems to be to disrupt fixation bias, or in other words, to dislodge old ideas rather than to generate new ones.^22^, ^121^, ^122^, ^203^
12. Perspective taking, world modeling, and other forms of narrative cognition have been identified as contributors to creativity, yet to reconcile their contribution with the prevalent view of creativity as computational, they have been mischaracterized as representational, associative, data‐driven processes.^204^, ^205^, ^206^
13. The semantic/visual ideas and concepts generated by divergent thinking have been identified as mechanically distinct from the physical actions deployed in most real‐world cases of problem solving. Yet, current creativity training continues to promote divergent thinking as the ultimate source of problem solving.^20^, ^29^, ^49^, ^99^
14. Creativity researchers have observed the “enigma” of a gap between minor and major innovation—and have noted that the gap cannot be overcome by more divergent thinking.^63^, ^191^, ^201^, ^207^, ^208^, ^209^
15. Creativity has been linked to emotion and self‐efficacy, yet emotion and self‐efficacy are linked in the mind not with logic but with narrative.^27^, ^39^, ^82^, ^83^, ^92^, ^210^, ^211^, ^212^, ^213^, ^214^
16. Because divergent thinking is a computational process, it can be (and has been) automated via AI; yet, even though AI can execute divergent thinking at vastly greater scale and efficiency than humans, it has not shown improved aptitude at most creative tasks.^132^, ^161^
17. Original action is a major component of new business plans, technological innovations, military strategies, marketing campaigns, political platforms, commercial art (e.g., novels and films), personal biographies, and daily behaviors, yet narrative has been restricted in current creativity research to the selective, postgenerative phase of innovation.^29^, ^53^, ^215^, ^216^, ^217^

These lacunae and incongruities can be addressed, and in some cases fully resolved, by granting narrative cognition a role in the generation phase of creativity. That role explains why possibility thinking, which is deeply narrative in form, is effective at increasing creativity; identifies some of the currently mysterious neural functions involved in counterfactual thinking, causal learning, and practical creativity; expands creativity training to improve every skill assessed on the Torrance Test; explicates perspective taking and world modeling on their own terms; closes the gap between brainstorming and problem solving and between minor and major innovation; illuminates the creative mechanism through which science leaps ahead; reveals why data (which is nonessential for narrative and can quickly jam narrative processing) have such an apparently peculiar relationship with creativity; integrates emotion and self‐efficacy into the mechanics of creative production; explains the creative limits of computer AI; and provides a direct path to increasing the creative production of business plans, technological mechanics, military strategies, marketing campaigns, political platforms, novels and films, and so on.

To grant narrative cognition a role in creativity is also to address the recent experimental finding that training in divergent thinking can boost aptitude at divergent thinking without leading to downstream improvements in problem solving or innovation.^31^, ^92^, ^100^, ^105^, ^218^, ^219^, ^220^, ^221^, ^222^ This finding is congruent with the discovery that computer AI can scale divergent thinking without scaling problem solving or innovation, and it potentially undercuts the value of current creativity training by inviting the charge that the training is a circular activity that produces measurable improvements in an inapplicable skill. By expanding brainstorming and other established training activities to target narrative processes, however, it becomes possible to acknowledge that divergent thinking might be insufficient for creative generation without diminishing the overall place of a creativity‐focused curriculum within schools, corporations, and government sectors.

And finally, irrespective of the merits of current creativity training, an attention to narrative cognition offers a potential way to address the public concern that student creativity is dropping at the same moment that demand for creativity is increasing among businesses and government organizations.^13^, ^197^, ^223^, ^224^, ^225^, ^226^, ^227^, ^228^, ^229^, ^230^, ^231^, ^232^, ^233^ There is a vast and growing appetite for creativity in the public marketplace,^37^, ^234^, ^235^, ^236^, ^237^, ^238^, ^239^ and a new curriculum rooted in narrative techniques, such as world building, perspective shifting, and action generation, can help respond to that appetite by offering a scientific and assessable training method with the potential to be as diverse, expansive, and psychologically nourishing as humanity's global library of novels, films, and other story‐based art. Even for scholars, trainers, and applied researchers who remain wary of narrative theory's broader theoretical claims (e.g., that narrative cognition cannot be reduced to computational processes), this new method affords a practical way to provide students with creativity training that extends beyond the established curriculum of divergent thinking.

## Conclusion

As creativity research has demonstrated, outsider perspectives can innovate inquiry and practice by seeing past accultured blind spots and expert bias.^26^, ^202^, ^240^, ^241^, ^242^, ^243^ In the case of creativity research, one such outsider perspective is narrative theory. Although narrative theory has not previously been applied to creativity research, it is a major, long‐established, and well‐respected field of academic inquiry that can:

1. Offer a rigorously mechanistic and empirical approach to creativity research that preserves J.P. Guilford's great breakthrough of demystifying creativity so that it can be programmatically trained and assessed.
2. Extend beyond Guilford's computational model of cognition to more fully account for the observed creative potential of the human mind and explain the observed creative limits of current artificial intelligences.
3. Resolve gaps and inconsistencies in current creativity theory, including the fact that logical mental processes, such as divergent thinking, cannot account for the creative activity of young children, thereby offering remedy for (a) the marginalization of creativity researchers (e.g., the followers of Anna Craft) who specialize in child creativity; (b) the splitting of the field between experts in adult creativity (who consult for the military and major corporations, exerting heavy influence over the hugely lucrative arena of professional creativity training) and experts in child creativity (who have been largely relegated to a small advisory role in early education); and (c) the missed opportunity to systematically extend the insights gained from studying child creativity onto adult populations.

And importantly, narrative theory's contribution to creativity research extends out of the theoretical into the practical. Narrative theory not only identifies the operational limits of divergent thinking and promotes existing methods for nurturing young children's creativity but also generates a diverse suite of new and assessable creativity training techniques. A great deal of future research is required to measure the effectiveness and cost–benefits of these techniques, but some indication that their creativity training is both genuinely new and genuinely needed—filling important holes in existing practice—has been provided by their early adoption by expert instructors in the U.S. military special operations community and other government, corporate, graduate, and professional programs.

None of these new narrative techniques invalidate Guilford's 70‐year‐old curriculum of divergent thinking or its use of semantic, pattern‐driven methods, such as association, transposition, conceptual blending, metaphor, extension, and redefinition. They simply address the empirical finding that creativity can extend beyond computation into the action‐based neural processes that evolved to help animal life adapt to the uncertainty and volatility of contested domains. Such processes continue to drive young children's “possibility thinking” and remain central to adult strategy, technology, science, narrative art, and everyday dreaming. For organizations and individuals who want to make those processes more generative, narrative theory affords a rich corpus of mechanical insights that can potentially be developed into innovative curricula and assessments for providing more of creativity's known social and personal benefits, from increased GDP, national security, and topline revenue to greater psychological resilience, positive affect, and personal growth.

## Competing interests

The authors are, as described herein, actively advising organizations, public and private, domestic, and international, including the U.S. government, on creativity training and so potentially stand to benefit financially and/or reputationally. To help divest interest, the authors declare all training described herein as public domain. All views expressed are the authors’ own.

### Peer review

The peer review history for this article is available at: https://publons.com/publon/10.1111/nyas.14763

## Footnotes

a Because our curriculum is still in its pilot stages, we cannot provide data on its practical yield. We hope to begin publishing such data in the near future, following scheduled randomized control trials in 2022–23, headed by Dr. Richard McConnell of the Command and General Staff College, on more than 600 U.S. Army majors. As a preliminary indication of the training's effectiveness, however, we offer the following expert testimony (which has generally proven to be as reliable as other existing assessments of creativity training^166^, ^167^, ^168^). Our training has been deemed “critical” by U.S. Army special operation trainers and “invaluable for training creative strategy” by Kenneth Long, Associate Professor at the Command and General Staff College.^169^ Within Fortune 50 companies, our training has been endorsed by senior executives at corporations, such as State Farm, as a way to “trigger great ideas,” “think differently,” and “identify other options.” And within graduate and continuing education, the training has been described by Richard Sugarman, Head of the Department of Systems and Software Engineering Management at the Air Force Institute of Technology, as “an outstanding tool for creative problem‐solving and innovation,” by Gregory D. Bunch, professor of Entrepreneurship at the University of Chicago Booth School of Business, as “a potent source of creative strategy,” and by Ann D. Christy, Professor of Engineering Education at the Ohio State University as a method that “revolutionizes the teaching of engineering design skills and enhances students’ professional development and career readiness.”
